# Androgen‐Driven Dysregulation of Lipolysis in Cumulus Cells: Molecular Evidence and Metabolic Implications in Polycystic Ovary Syndrome

**DOI:** 10.1002/rmb2.70093

**Published:** 2026-08-31

**Authors:** Reza Zarezadeh, Amir Fattahi, Kobra Hamdi, Saba Nikanfar, Mohammad Nouri, Masoud Darabi

**Affiliations:** ^1^ Women's Reproductive Health Research Center Tabriz University of Medical Sciences Tabriz Iran; ^2^ Department of Biochemistry and Clinical Laboratories, Faculty of Medicine Tabriz University of Medical Sciences Tabriz Iran; ^3^ Research Pole in Reproductive Physiopathology, Institute of Experimental and Clinical Research Catholic University of Louvain Brussels Belgium; ^4^ Stem Cell Research Center Tabriz University of Medical Sciences Tabriz Iran; ^5^ University of Lübeck, Department of Hematology and Oncology University Cancer Center Schleswig‐Holstein (UCCSH) and University Hospital Schleswig‐Holstein (UKSH), Campus Lübeck Lübeck Germany

**Keywords:** granulosa cells, hormones, in vitro fertilization, lipid metabolism, ovarian diseases

## Abstract

**Objective:**

The present study aimed to investigate lipolytic function in cumulus cells (CCs) from women with polycystic ovary syndrome (PCOS), with a particular emphasis on the role of androgen excess.

**Study Design:**

Samples of follicular fluid and CCs were collected from 30 PCOS and 30 control subjects. Human primary CCs were cultured and treated with high doses of testosterone. Androgen and gene expression levels were measured by chemiluminescent immunoassay and quantitative reverse transcription polymerase chain reaction, respectively. Oil Red O staining followed by spectrophotometric analysis was conducted to quantify intracellular neutral lipids.

**Main Findings:**

CCs from PCOS patients showed significantly downregulated *PNPLA2* and elevated *G0S2* and *HILPDA* expression, with concomitant increased lipid droplet content (*p* < 0.05). The multivariable logistic regression model constructed from the combination of all three genes exhibited a superior diagnostic power (AUC = 0.837, *p* < 0.001). Only *HILPDA* expression was significantly correlated with intrafollicular testosterone (*r* = 0.395, *p* = 0.031), yet the main alterations observed in PCOS CCs were recapitulated in normal cells exposed to high doses of testosterone (*p* < 0.05).

**Conclusion:**

PCOS‐associated hyperandrogenism appears to impair lipolysis in CCs, representing a potential mechanistic contributor to attendant compromised oocyte competence.

**Abbreviations**
ACC1: acetyl‐CoA carboxylase 1
ANOVA: analysis of variance
ATGL: adipose triglyceride lipase
AUC: area under the curve
BMI: body mass index
CC: cumulus cell
CGI‐58: comparative gene identification‐58
CI: confidence interval
CREB: cAMP‐response element binding protein
DHEA‐S: dehydroepiandrosterone sulfate
DMEM: dulbecco's Modified Eagle Medium
ELISA: enzyme‐linked immunosorbent assay
F12: ham's F‐12 Nutrient Mixture
FASN: fatty acid synthase
FBS: fetal bovine serum
FF: follicular fluid
FOXO1: forkhead box protein O1
FSH: follicle‐stimulating hormone
G0S2: G0/G1 switch 2
GAPDH: glyceraldehyde‐3‐phosphate dehydrogenase
GC: granulosa cell
GnRH: gonadotropin‐releasing hormone
hCG: human chorionic gonadotropin
HIF: hypoxia‐inducible factors
HILPDA: hypoxia‐inducible lipid droplet associated
ICSI: intracytoplasmic sperm injection
IVF: in vitro fertilization
LH: luteinizing hormone
NPV: negative predictive value
OR: odds ratio
PBS: phosphate‐buffered saline
PCOS: polycystic ovary syndrome
PPAR: peroxisome proliferator‐activated receptor
PPV: positive predictive value
qPCR: quantitative real‐time polymerase chain reaction
ROC: receiver operating characteristic
SD: standard deviation
SREBP1: sterol regulatory element‐binding protein 1
ZEB1: zinc finger E‐box binding homeobox 1

## 1 Introduction

Polycystic ovary syndrome (PCOS) is a common endocrine disorder that affects approximately 5%–18% of women worldwide during childbearing years, depending on the criteria used for diagnosis, and underlies most cases of anovulatory infertility. Recognized typically by androgen excess, oligomenorrhea or amenorrhea, and numerous cysts visible on ovarian ultrasound, PCOS results in intricate endocrine, metabolic, and reproductive consequences, underscoring its multidimensional nature. Apart from the deleterious impact on fertility capacity, PCOS has a close relationship with multiple metabolic aberrations, including obesity, insulin resistance, hyperlipidemia, and metabolic syndrome. Moreover, it predisposes affected women to type 2 diabetes mellitus, cardiovascular disorders, and malignancy, which highlights substantial long‐term health implications of PCOS [1]. Mounting evidence suggests that, beyond systemic metabolic disruptions, local metabolic derangements in the follicular milieu play a crucial role in the pathophysiology of PCOS, especially by influencing cumulus cells (CCs) and the oocyte [2].

CCs, in conjunction with mural granulosa cells (GCs), surround the oocyte within the developing follicle and are essential for providing energetic substrates, regulating glucose and lipid metabolism, and supporting meiotic maturation [3]. Metabolic dysfunction of CCs has been consistently reported in women with PCOS, including altered mitochondrial respiratory capacity, abnormal glycolysis, and defective lipid homeostasis [4]. Lipid metabolism, in particular, is one of the key metabolic pathways within different follicular constituents, which significantly contributes to the progression and regulation of folliculogenesis and can thereby profoundly impact oocyte maturation and developmental competence [5]. Emerging data from transcriptomic studies indicate that PCOS is associated with disturbed lipid metabolism in GCs [6, 7, 8]. As a defining characteristic of PCOS, hyperandrogenism seems to be actively implicated in these metabolic perturbations by promoting intracellular neutral lipid accumulation, altering lipid metabolism‐related gene expression, and impairing mitochondrial function in GCs [9, 10, 11]. One of the important subprocesses of lipid metabolism is lipolysis, in which triglycerides stored in lipid droplets are mobilized and broken down into glycerol and fatty acids. Experimental findings in PCOS animal models show that ovarian tissue contains excessive amounts of intracellular neutral lipids [12, 13, 14], potentially reflecting suppressed lipolytic activity in the ovaries. Nevertheless, direct evidence on whether PCOS, especially through androgen excess, alters lipolysis in human ovarian follicular cells is lacking thus far.

Canonical neutral lipolysis is comprised of three sequential reactions catalyzed by adipose triglyceride lipase (ATGL), hormone‐sensitive lipase, and monoglyceride lipase, respectively. ATGL, encoded by the *PNPLA2* gene, mediates the first and rate‐limiting step of the pathway. The full function of ATGL is achieved in the presence of a coactivator named comparative gene identification‐58 (CGI‐58), which is encoded by the *ABHD5* gene. Conversely, G0/G1 switch 2 (G0S2) and hypoxia‐inducible lipid droplet‐associated (HILPDA) are other peptide co‐regulators that serve as inhibitors of ATGL activity [15]. As crucial components of the lipolytic machinery, the status of these proteins is a key determinant of the lipolysis rate and, consequently, of lipid storage homeostasis and cellular energy balance. However, their function and regulation in human ovarian follicular cells, particularly under PCOS‐specific metabolic and endocrine conditions, remain poorly characterized.

The current study sought to explore the status of lipolysis in CCs from women with PCOS, with a specific focus on the effects of androgen excess. To this end, gene expression of *PNPLA2*, *ABHD5*, *G0S2*, and *HILPDA*, as major contributors to the pathway's regulatory step, along with intracellular neutral lipids as a surrogate biomarker of lipolytic activity, was assessed.

## 2 Materials and Methods

### 2.1 Study Population

A total of 60 non‐obese women, including 30 PCOS patients and 30 age‐ and body mass index (BMI)‐matched controls, were randomly recruited from individuals referred to the fertility and infertility center of Tabriz University of Medical Sciences to receive intracytoplasmic sperm injection (ICSI). PCOS was diagnosed based on the 2003 Rotterdam criteria, manifesting as at least two symptoms out of the following (1): amenorrhea or oligomenorrhea (2), clinical or biochemical androgen excess, and (3) ultrasound‐visualized polycystic ovarian morphology. The control group consisted of women with normal ovarian function who failed to conceive naturally due to their partners' infertility or tubal obstruction. The eligibility criteria for inclusion in the study were as follows: Age 20–40 years, BMI 18–29.9 kg/m^2^, undergoing the first in vitro fertilization (IVF) cycle, and adherence to a uniform ovarian stimulation protocol. In contrast, the exclusion criteria comprised endocrine abnormalities induced by diabetes mellitus, thyroid diseases, congenital adrenal hyperplasia, Cushing syndrome, hypogonadotropic hypogonadism, hyperprolactinemia, androgen‐secreting tumors, and premature ovarian insufficiency, as well as a history of hormonal or insulin‐sensitizing medication use during the last three months prior to enrollment.

### 2.2 Controlled Ovarian Stimulation and ICSI

Menstruation was achieved either spontaneously or through administration of low‐dose monophasic oral contraceptive pills for 21–25 days. Controlled ovarian stimulation was carried out using the gonadotropin‐releasing hormone (GnRH) antagonist protocol. In brief, daily doses of recombinant follicle‐stimulating hormone (Follitropin alfa; Cinnal‐F, CinnaGen, Karaj, Iran) and/or human menopausal gonadotropin (Menotropins; PDHoMoG, Pooyesh Darou, Tehran, Iran) were administered to all subjects starting on day 2 or 3 of the menstrual cycle. Thereafter, participants received the GnRH antagonist (Cetrorelix; Cetrotide, Serono, Geneva, Switzerland) at a dose of 0.125 mg per day from stimulation day 5 or 6 until the dominant follicle(s) reached ≥ 18 mm in diameter. Ovarian response was monitored by inspecting the number and size of follicles via ultrasound, as well as by quantifying plasma estradiol. Upon reaching a diameter of ≥ 18 mm in the dominant follicle(s), ovulation was triggered by a 10 000 IU dose of human chorionic gonadotropin (hCG; Pregnyl, Organon, Oss, Netherlands). Cumulus‐oocyte complexes were aspirated 36 h later through transvaginal ultrasound‐guided follicular puncture. After mechanical removal of surrounding CCs, the ICSI technique was used to inseminate mature oocytes with a single motile sperm. The observation of two pronuclei and polar bodies by microscopy 16–18 h post‐insemination was considered the confirmation of successful oocyte fertilization. Two fresh cleaved embryos were transferred to the patients’ uteri under ultrasound guidance 48 h after ICSI. Clinical pregnancy was established through an elevation of plasma β‐hCG to higher than 25 mIU/mL, followed by the observation of a gestational sac in the uterus by ultrasound 14 days after embryo transfer. The fertilization rate was defined as the proportion of fertilized oocytes to MII oocytes.

### 2.3 Collection of Follicular Fluid and CCs

At the time of oocyte aspiration, follicular fluid (FF) samples without blood contamination were obtained from mature follicles (≥ 18 mm in diameter). The specimens were subsequently centrifuged at 1 500 g for 10 min, and the resultant supernatants were retrieved and stored at −80°C for future experiments. CCs were derived from mechanical denudation of oocytes via needles. Isolated CCs were collected in 1.5 mL tubes afterwards and centrifuged at 500 g for 5 min. The cell pellet was then washed with phosphate‐buffered saline (PBS) and stored at −80°C for gene expression analysis or otherwise resuspended in culture medium for in vitro culture.

### 2.4 Cell Culture and Treatment

The isolated and pooled CCs from non‐PCOS participants were cultured in Dulbecco's Modified Eagle Medium/Ham's F‐12 Nutrient Mixture (DMEM/F12) medium (Gibco, Thermo Fisher Scientific, Waltham, MA, USA) supplemented with 10% fetal bovine serum (FBS; Gibco, Thermo Fisher Scientific, Waltham, MA, USA) and 1% penicillin–streptomycin antibiotics (10,000 IU/mL stock, Gibco, Thermo Fisher Scientific, Waltham, MA, USA) at 37°C under a humidified atmosphere containing 5% CO_2_ for 3–5 days. CCs were thereafter seeded into 6‐well plates at a density of 2 × 10^5^ cells per well and cultured in the same conditions. The next day, cells were treated with 5, 10, or 20 ng/mL testosterone (Merck, Darmstadt, Germany) or solvent in a culture medium containing 2% FBS for 48 h. Treatment concentrations were selected based on the range of testosterone concentrations observed in FF from women with and without PCOS.

### 2.5 Biochemical Analysis

Enzymatic colorimetric assays (Pars Azmun, Tehran, Iran) were performed on a Hitachi 717 chemistry analyzer (Hitachi, Tokyo, Japan) to measure concentrations of glucose, triglycerides, and total cholesterol. Insulin, follicle‐stimulating hormone (FSH), luteinizing hormone (LH), total testosterone, and dehydroepiandrosterone sulfate (DHEA‐S) levels were quantified by an Immulite 2000 XPi chemiluminescent immunoassay system (Siemens Healthineers, Erlangen, Germany) with analytical sensitivities of 2 μIU/mL, 0.1 mIU/mL, 0.05 mIU/mL, 15 ng/dL, and 3 μg/dL, respectively, following the manufacturer's instructions. An enzyme‐linked immunosorbent assay (ELISA) kit (Elabscience, Houston, TX, USA; detection limit, 18.75 pg/mL) was used to determine concentrations of free testosterone.

### 2.6 RNA Isolation and Quantitative Reverse Transcription Polymerase Chain Reaction

Total RNA was isolated from CCs using YTzol reagent (Yekta Tajhiz Azma, Tehran, Iran) according to the manufacturer's protocol. RNA concentration and purity were determined by measuring optical density at 260 nm and 260/280 and 260/230 ratios, respectively, via a NanoDrop ND‐1000 spectrophotometer (NanoDrop Technologies, Wilmington, DE, USA). A total of 1 μg RNA was reverse transcribed into cDNA using a cDNA synthesis kit (Yekta Tajhiz Azma, Tehran, Iran) at 42°C for 1 h, followed by enzyme inactivation at 70°C for 5 min.

Quantitative real‐time polymerase chain reaction (qPCR) was carried out using SYBR Green qPCR Master Mix (Yekta Tajhiz Azma, Tehran, Iran) on a Mic qPCR cycler (Bio Molecular Systems, Queensland, Australia). Amplification was continued for 40 cycles, each of which consisted of three phases as follows: denaturation at 95°C for 15 s, annealing at the primer‐specific optimized temperature for 30 s, and extension at 72°C for 30 s. Gene expression was analyzed using the 2‐^ΔΔCt^ method, normalized to glyceraldehyde‐3‐phosphate dehydrogenase (GAPDH) as a reference gene. The sequences of primer pairs are listed in Table S1.

### 2.7 Oil Red O Staining

Cell slides were washed three times with PBS and then fixed using 4% paraformaldehyde at room temperature for 30 min. A working solution of 0.3% Oil Red O was prepared by adding 4 mL of distilled water to 6 mL of 0.5% Oil Red O stock solution (Merck, Darmstadt, Germany), followed by filtration through filter paper. After washing thrice with PBS, the fixed CCs were stained using the working solution of Oil Red O at room temperature for 30 min, washed three times with PBS, and photographed under a Cytation 5 microscope (BioTek Instruments, Winooski, VT, USA) in the phase contrast channel. For the purpose of quantifying lipid droplet content, Oil Red O dye was extracted from the completely dried stained cells using 100% isopropanol, and its optical density was measured at a wavelength of 510 nm via a BioTek 800 TS microplate reader (BioTek Instruments, Winooski, VT, USA).

### 2.8 Statistical Analysis

All statistical analyses were performed using SPSS Statistics 26.0 (IBM SPSS Inc., Chicago, IL, USA) and GraphPad Prism 9 (GraphPad Software, San Diego, CA, USA). The one‐sample Kolmogorov–Smirnov test was used to examine the normality of data distribution in the case of quantitative variables. Data were presented as mean ± standard deviation (SD), median (interquartile range), and frequency (percentage frequency), depending on the scale and distribution type of variables. Numerical variables with normal and non‐normal distributions were compared between women with PCOS and non‐PCOS controls by the independent samples *t*‐test and Mann–Whitney U test, respectively. The chi‐squared (*χ* ^2^) test was used to compare the groups in terms of the sole categorical variable, namely pregnancy rate. Binomial logistic regression was utilized to determine the association, specifically the odds ratio (OR) and 95% confidence interval (CI), of variables with the risk of PCOS. The adjustment for possible confounding factors was made by including them as covariates in the logistic regression models. The potential of variables for the diagnosis of PCOS was evaluated by the receiver operating characteristic (ROC) analysis. Pearson's correlation, Spearman's rank correlation, and linear regression analyses were employed to identify possible correlations between quantitative variables. Different treatment groups were compared by the one‐way analysis of variance (ANOVA), followed by Tukey's post hoc test. A *p*‐value of < 0.05 was regarded as statistically significant.

## 3 Results

### 3.1 Baseline Characteristics of the Participants

Demographic, biochemical, and clinical characteristics of the study population are summarized in Table 1. Age and BMI did not differ statistically between women with PCOS and controls (*p* > 0.05). Significant intergroup differences were observed in intrafollicular concentrations of glucose (*p* < 0.001), triglycerides (*p* = 0.007), FSH (*p* = 0.002), LH (*p* < 0.001), total testosterone (*p* = 0.003), and DHEA‐S (*p* = 0.002), all of which were higher in the PCOS group. Free testosterone exhibited a trend toward elevation in PCOS patients, although it did not reach statistical significance (*p* = 0.059). No significant differences were found in terms of FF total cholesterol or insulin levels between the two groups (*p* > 0.05).

**TABLE 1:** Baseline characteristics of the participants.

	Control	PCOS	*p*
No.	30	30	
Age, years	30.57 ± 5.21	30.37 ± 4.51	0.874^a^
BMI, kg/m^2^	25.82 ± 3.12	25.71 ± 3.34	0.899^a^
Glucose, mg/dL	51.00 (40.75–58.00)	62.00 (58.75–74.75)	< 0.001
Triglyceride, mg/dL	8.73 ± 3.28	12.00 ± 5.46	0.007^a^
Cholesterol, mg/dL	33.77 ± 10.53	30.97 ± 10.26	0.301^a^
Insulin, μIU/mL	3.80 (1.37–5.90)	4.20 (1.38–16.45)	0.308
FSH, mIU/mL	5.15 (3.58–6.92)	8.55 (5.18–9.78)	0.002
LH, mIU/mL	2.90 (1.92–10.88)	15.40 (7.80–30.78)	< 0.001
Total testosterone, ng/mL	5.65 (4.38–8.65)	8.10 (6.22–16.80)	0.003
Free testosterone, pg/mL	7.10 (5.25–11.00)	8.25 (6.65–16.32)	0.059
DHEA‐S, μg/dL	106.87 ± 50.20	167.37 ± 88.26	0.002^a^
Gonadotropin dose, IU	1594.67 ± 434.65	1316.00 ± 346.26	0.008^a^
MII oocytes, *n*	8.50 (6.00–13.00)	16.00 (9.75–18.25)	0.001
Cleaved embryos, *n*	6.50 (5.00–11.00)	11.00 (9.00–15.00)	0.013
Fertilization rate, %	88.75 (77.58–100.00)	76.4 (59.70–88.38)	0.005
Pregnancy, *n* (%)	11 (36.67)	10 (33.33)	0.787^b^

*Note:* Data are presented as mean ± SD, median (Q1‐Q3), or frequency (percentage). *p* values represent the significance level of the Mann–Whitney U test unless otherwise indicated.

Abbreviations: BMI, body mass index; DHEA‐S, dehydroepiandrosterone sulfate; FSH, follicle‐stimulating hormone; LH, luteinizing hormone.

^a^ Independent samples *t*‐test.

^b^ Chi‐square (χ^2^) test.

With regard to IVF outcomes, women with PCOS demonstrated statistically higher numbers of retrieved mature oocytes (*p* = 0.001) and cleaved embryos (*p* = 0.013) compared to controls. Conversely, the total gonadotropin dose administered for ovarian stimulation and the fertilization rate were significantly lower in the PCOS group (*p* = 0.008 and *p* = 0.005, respectively). Finally, the clinical pregnancy rate was comparable between PCOS and control participants (*p* > 0.05).

### 3.2 Dysregulation of Lipolysis in CCs From Women With PCOS

**FIGURE 1:**
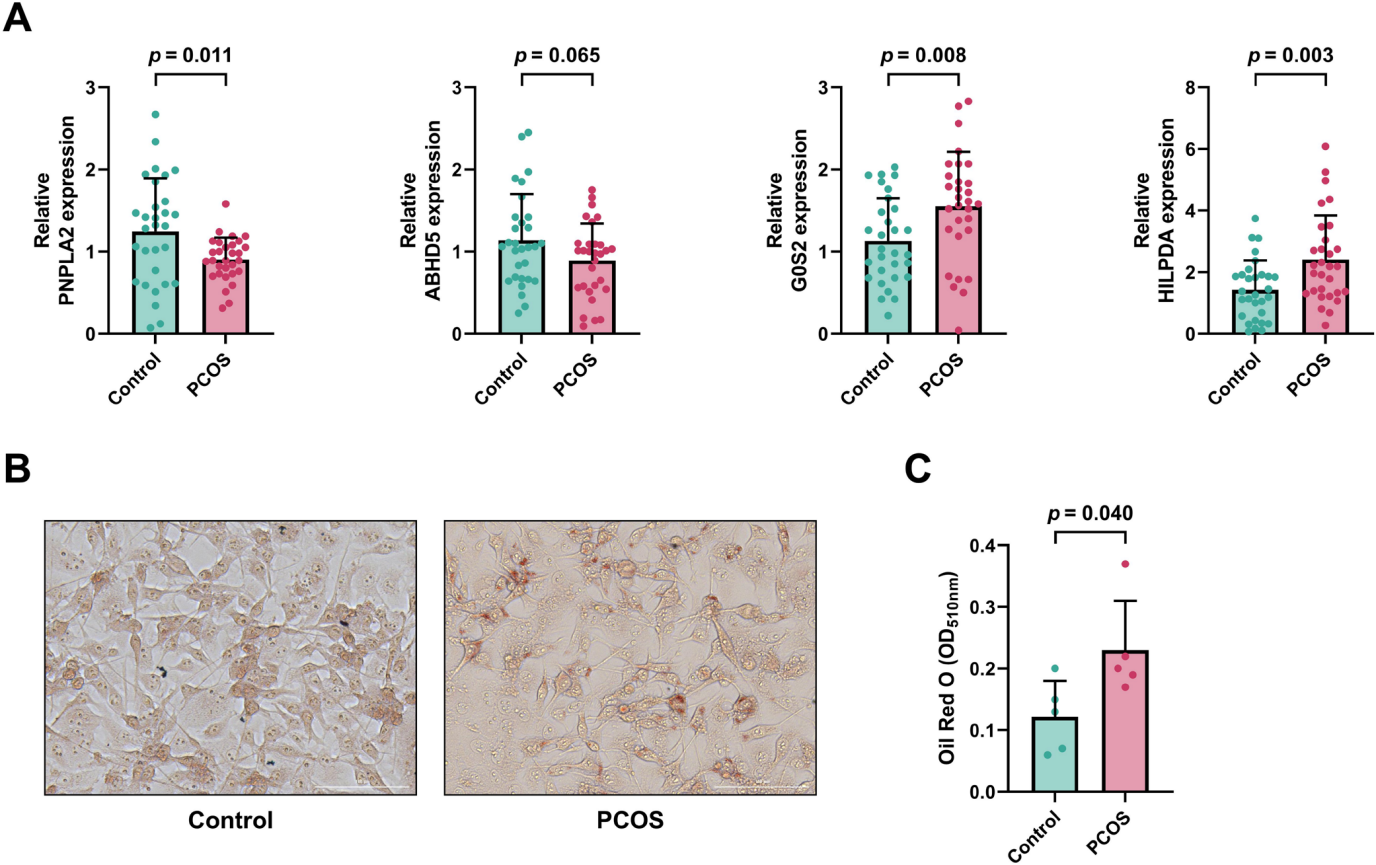
Dysregulation of lipolysis in CCs from women with PCOS. (A) Expression levels of the lipolysis‐related genes in CCs from control and PCOS subjects, assessed by RT‐qPCR (*n* = 30 per group). (B) Representative images and (C) lipid droplet content in CCs from control and PCOS subjects, visualized by Oil Red O staining and quantified by measuring the optical density of the extracted dye at 510 nm (*n* = 5 per group). Scale bar = 100 μm. Data are presented as mean ± SD. *p*‐values represent the statistical significance of the independent‐samples *t*‐test.

Gene expression of *PNPLA2*, *ABHD5*, *G0S2*, and *HILPDA*, together with intracellular neutral lipids, was assessed in CCs from the subjects. *PNPLA2* expression was statistically reduced in PCOS women compared to controls (*p* = 0.011), whereas *G0S2* and *HILPDA* were significantly upregulated (*p* = 0.008 and *p* = 0.003, respectively; Figure 1A). Although mRNA levels of *ABHD5* displayed a decreasing trend in the PCOS group, the difference remained statistically insignificant (*p* = 0.065; Figure 1A). Lipid droplet accumulation was elevated in CCs from PCOS patients relative to controls (*p* = 0.040; Figure 1B,C).

### 3.3 Potential of the Lipolysis‐Related Genes for Diagnosis of PCOS

To evaluate the association between expression of the lipolysis‐related genes in CCs and the risk of PCOS, logistic regression analyses were performed for each gene individually (single‐gene models), both before and after adjustment for the confounding factors age and BMI (Table 2 and Table S2). In unadjusted analyses, *PNPLA2* downregulation was significantly associated with higher odds of PCOS (OR [95% CI] = 0.225 [0.067, 0.757], *p* = 0.016). This association remained robust after adjusting for age and BMI (*p* = 0.014). Similarly, *G0S2* was positively associated with the likelihood of PCOS (OR [95% CI] = 3.308 [1.289, 8.486], *p* = 0.013), and the association persisted after adjustment (*p* = 0.011). *HILPDA* upregulation also showed a significant association with higher odds of PCOS (OR [95% CI] = 2.075 [1.219, 3.532], *p* = 0.007), which was independent of age and BMI (*p* = 0.006). On the contrary, *ABHD5* only tended to be inversely associated with the likelihood of PCOS, but the association did not reach statistical significance (OR [95% CI] = 0.370 [0.125, 1.092], *p* = 0.072).

**TABLE 2:** Logistic regression analysis of the lipolysis‐related genes in association with the odds of PCOS.

	Unadjusted			Adjusted for age and BMI	
	OR [95% CI]	*p*		OR [95% CI]	*p*
*Single‐gene models*					
PNPLA2	0.225 [0.067, 0.757]	0.016		0.210 [0.060, 0.733]	0.014
*R* ^2^ = 0.149			*R* ^2^ = 0.157		
ABHD5	0.370 [0.125, 1.092]	0.072		0.357 [0.118, 1.081]	0.068
*R* ^*2*^ = 0.077			*R* ^2^ = 0.079		
G0S2	3.308 [1.289, 8.486]	0.013		3.427 [1.322, 8.886]	0.011
*R* ^2^ = 0.150			*R* ^2^ = 0.156		
HILPDA	2.075 [1.219, 3.532]	0.007		2.255 [1.268, 4.010]	0.006
*R* ^2^ = 0.199			*R* ^2^ = 0.214		
*Multi‐gene model*					
PNPLA2	0.141 [0.036, 0.559]	0.005		0.132 [0.032, 0.555]	0.006
G0S2	4.548 [1.470, 14.070]	0.009		4.944 [1.492, 16.388]	0.009
HILPDA	2.285 [1.153, 4.528]	0.018		2.214 [1.084, 4.520]	0.029
*R* ^2^ = 0.455			*R* ^2^ = 0.459		

*Note:* *R* ^2^ values represent Nagelkerke's pseudo *R* ^2^.

Abbreviations: BMI, body mass index; CI, confidence interval; OR, odds ratio.

According to the results of the unadjusted multivariable logistic regression analysis (multi‐gene model), expression of each of *PNPLA2* (OR [95% CI] = 0.141 [0.036, 0.559], *p* = 0.005), *G0S2* (OR [95% CI] = 4.548 [1.470, 14.070], *p* = 0.009), and *HILPDA* (OR [95% CI] = 2.285 [1.153, 4.528], *p* = 0.018) in CCs emerged as an independent predictor of PCOS. Adjusting for age and BMI did not statistically eliminate the associations between transcript abundance of genes and the odds of PCOS in the multi‐gene model (*p* < 0.05).

**FIGURE 2:**
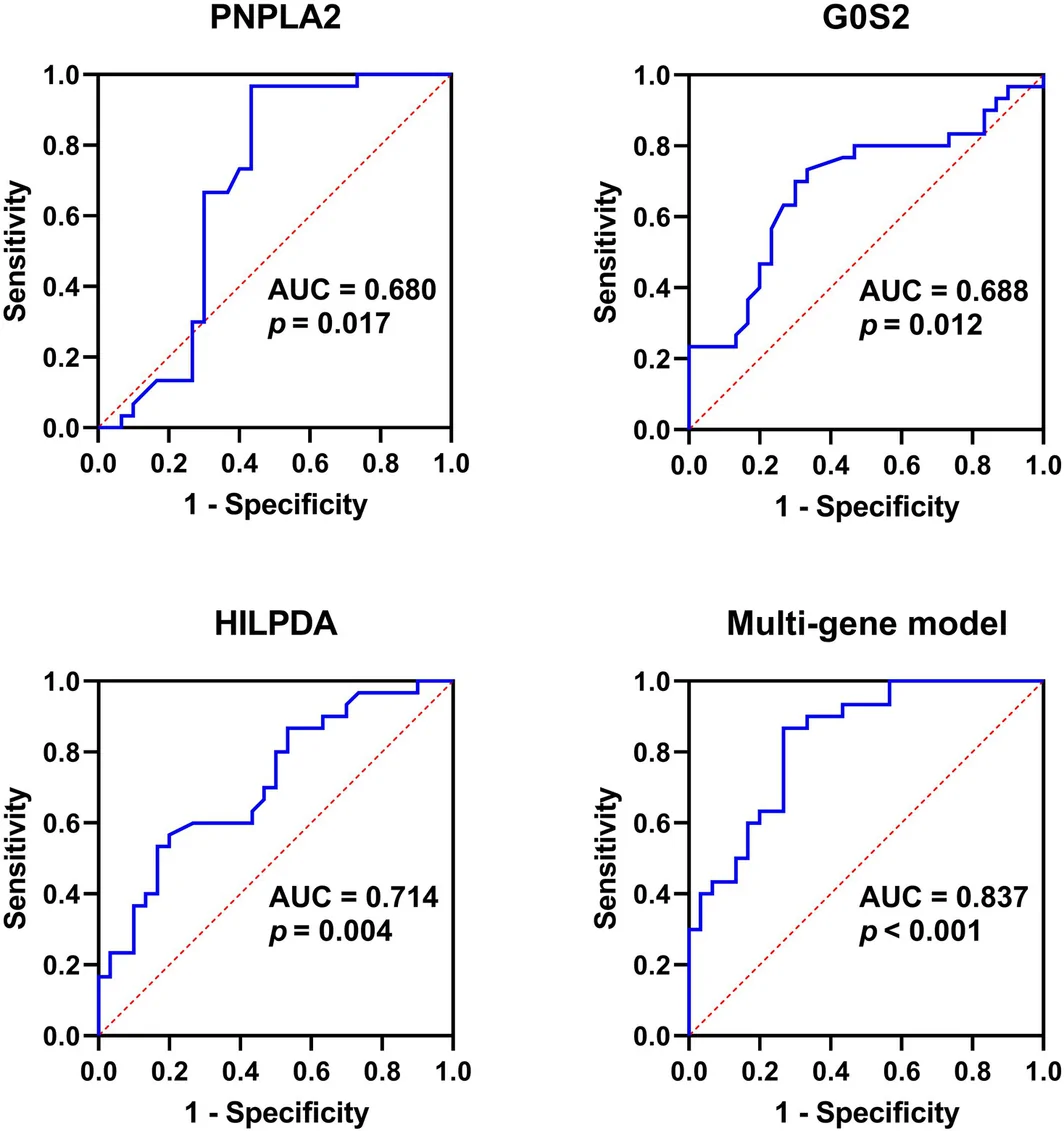
Potential of the lipolysis‐related genes for diagnosis of PCOS. ROC curves depicting the performance of the individual genes and the multi‐gene model to discriminate women with PCOS from controls. The multi‐gene model consists of *PNPLA2*, *G0S2*, and *HILPDA* as independent variables.

ROC curve analysis was employed to assess the performance of the statistically significant single‐gene and multi‐gene models in discriminating PCOS patients from controls. Accordingly, expression of all three individual genes, namely *PNPLA2*, *G0S2*, and *HILPDA*, displayed significant but moderate diagnostic power (*p* < 0.05; Figure 2). Among the single markers, *HILPDA* revealed the highest area under the curve (AUC = 0.714, *p* = 0.004), followed by *G0S2* (AUC = 0.688, *p* = 0.012) and *PNPLA2* (AUC = 0.680, *p* = 0.017). The multi‐gene model, which was constructed from the combination of *PNPLA2*, *G0S2*, and *HILPDA* as independent variables, demonstrated an excellent discriminatory efficiency between PCOS patients and control subjects with an AUC of 0.837 (*p* < 0.001). At the optimal cutoff, the model achieved a sensitivity of 86.67%, a specificity of 73.33%, a positive predictive value of 76.47%, and a negative predictive value of 84.62% (Table 3).

**TABLE 3:** Performance metrics of ROC analysis for the lipolysis‐related genes with respect to the diagnosis of PCOS.

	AUC	Cutoff	Sensitivity (%)	Specificity (%)	PPV (%)	NPV (%)	*p*
PNPLA2	0.680	1.260	96.67	56.67	69.05	94.45	0.017
G0S2	0.688	1.265	73.33	66.67	68.75	71.43	0.012
HILPDA	0.714	1.940	56.67	80.00	73.91	64.87	0.004
Multi‐gene model	0.837	−0.270	86.67	73.33	76.47	84.62	< 0.001

*Note:* The multi‐gene model consists of *PNPLA2*, *G0S2*, and *HILPDA* as independent variables.

Abbreviations: AUC, area under the curve; NPV, negative predictive value; PPV, positive predictive value.

### 3.4 Correlation of the Lipolysis‐Related Genes With Metabolic, Endocrine, and Reproductive Parameters

**FIGURE 3:**
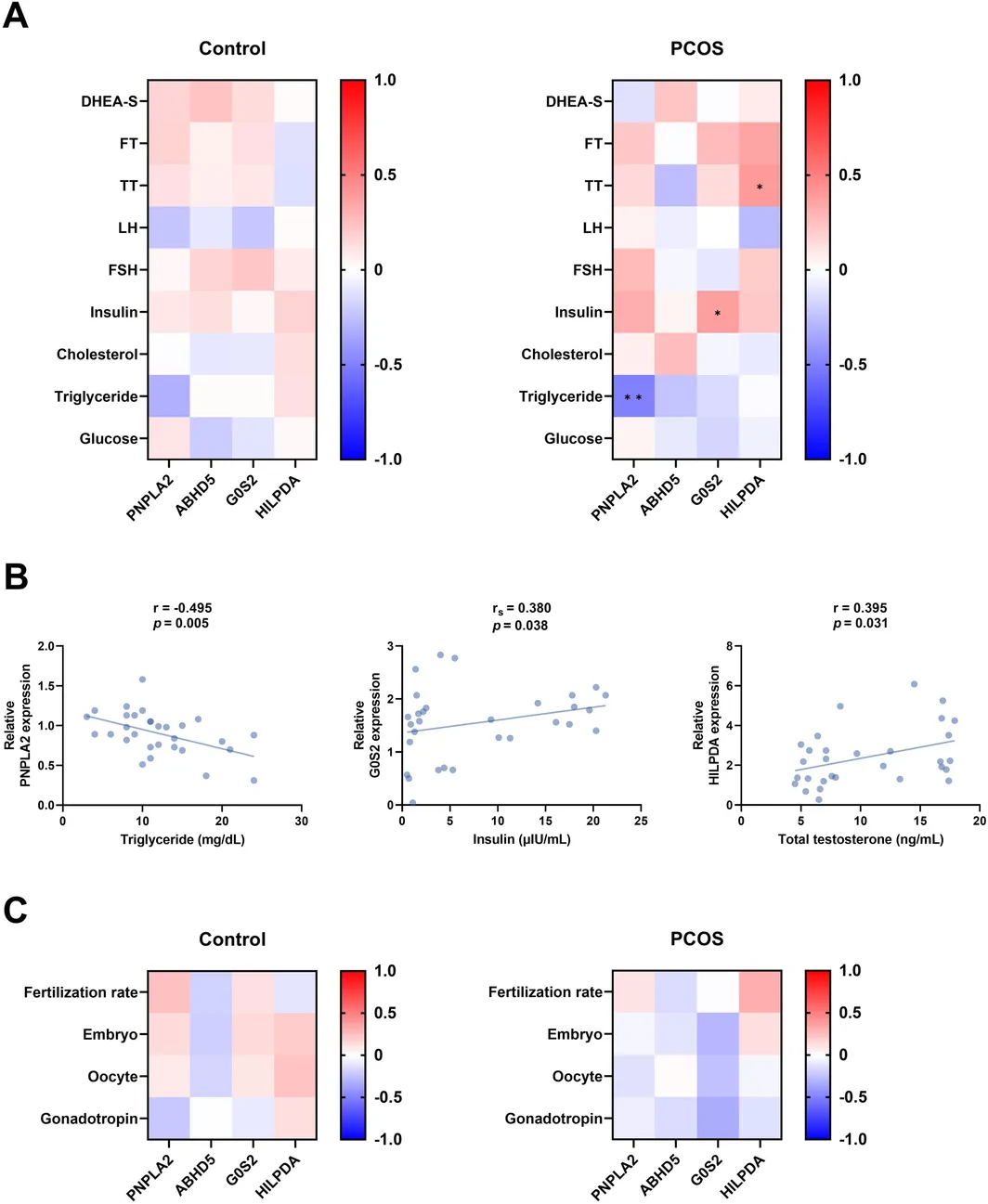
Correlation of the lipolysis‐related genes with metabolic, endocrine, and reproductive parameters. (A) Correlations between gene expression levels and metabolic and endocrine indices in the control and PCOS groups. (B) Statistically significant correlations from panel A. (C) Correlations between gene expression levels and reproductive outcomes in the control and PCOS groups. The r and r_s_ values represent Pearson's correlation coefficient and Spearman's rank correlation coefficient, respectively.

To explore whether expression of the lipolysis‐related genes in CCs is associated with intrafollicular metabolic and endocrine parameters, as well as reproductive outcomes, the correlation analysis was conducted separately in the PCOS and control groups. While none of the investigated genes showed a significant association in the control cohort, distinct statistical correlations were observed in the PCOS group (Figure 3A). Notably, mRNA abundance of *PNPLA2* was negatively correlated with FF triglyceride levels (*r* = −0.459, *p* = 0.005). Furthermore, a positive correlation was identified between *G0S2* and insulin (*r*_s_ = 0.380, *p* = 0.038), as well as between *HILPDA* and total testosterone (*r* = 0.395, *p* = 0.031; Figure 3B). With regard to IVF outcomes, no significant correlation with gene expression was noted in either the control or the PCOS group (*p* > 0.05; Figure 3C).

### 3.5 Testosterone‐Induced Dysregulation of Lipolysis in CCs

**FIGURE 4:**
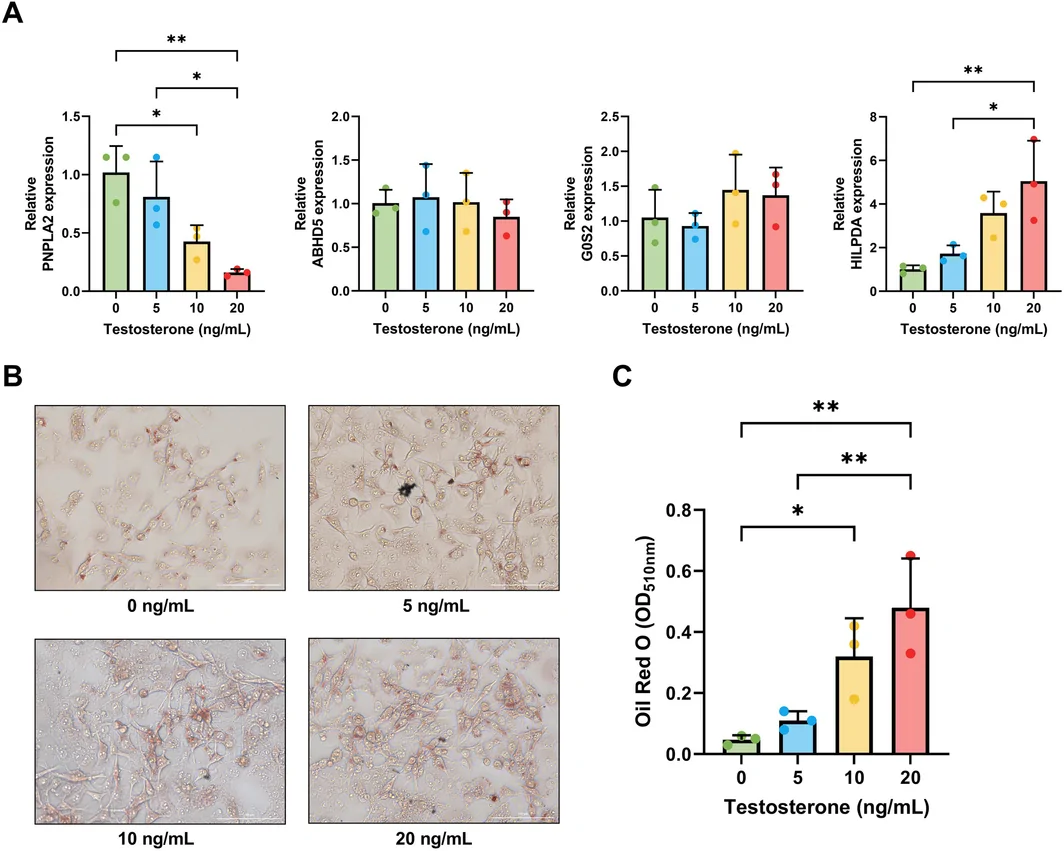
Testosterone‐induced dysregulation of lipolysis in cultured human CCs. (A) Expression levels of the lipolysis‐related genes in CCs treated with varying concentrations of testosterone, assessed by RT‐qPCR (*n* = 3 per group). (B) Representative images and (C) lipid droplet content in CCs treated with varying concentrations of testosterone, visualized by Oil Red O staining and quantified by measuring the optical density of the extracted dye at 510 nm (*n* = 3 per group). Scale bar = 100 μm. Data are presented as mean ± SD. *p*‐values represent the statistical significance of Tukey's post hoc test. *p < 0.05; **p < 0.01.

To determine if androgen excess contributes to the dysregulation of lipolysis in CCs of women with PCOS, expression of the lipolysis‐related genes and intracellular neutral lipid content in cultured human CCs were examined following treatment with varying concentrations of testosterone (0, 5, 10, and 20 ng/mL) for 48 h. Transcript levels of *ABHD5* and *G0S2* remained unaltered across all testosterone treatment groups (*p* > 0.05), whereas those of *PNPLA2* and *HILPDA* demonstrated a dose‐dependent trend in response to testosterone (*p* < 0.05; Figure 4A). In particular, *PNPLA2* was significantly downregulated in CCs treated with 10 ng/mL testosterone compared to unexposed controls (*p* = 0.029). This suppressive effect was further pronounced at a concentration of 20 ng/mL, which resulted in a marked reduction of *PNPLA2* relative to both the control and 5 ng/mL treatment groups (*p* = 0.004 and *p* = 0.018, respectively). Conversely, *HILPDA* was significantly upregulated in CCs treated with 20 ng/mL testosterone compared with cells exposed to 0 and 5 ng/mL testosterone (*p* = 0.008 and *p* = 0.022, respectively). Additionally, incubation with a high dose of testosterone (20 ng/mL) resulted in a dramatic elevation in lipid droplet accumulation relative to both the control and 5 ng/mL treatment groups (*p* = 0.004 and *p* = 0.010, respectively), while a moderate increase was also observed in cells treated with 10 ng/mL testosterone compared to the control (*p* = 0.047; Figure 4B,C).

## 4 Discussion

The current research aimed to provide insights into the status of lipolysis in CCs from women with PCOS, especially in relation to hyperandrogenism. Although earlier studies have shown that PCOS is accompanied by altered fatty acid levels in the oocyte microenvironment [16, 17, 18], whether this alteration reflects dysregulated lipolytic function in ovarian follicular cells has remained largely unexplored to date. In this study, we demonstrated that lipolysis was significantly inhibited in CCs from women with PCOS, characterized by decreased *PNPLA2* expression, upregulated *G0S2* and *HILPDA*, and elevated intracellular neutral lipids. These molecular alterations were associated with the likelihood of PCOS independent of BMI, with *PNPLA2*, *G0S2*, and *HILPDA* collectively exhibiting robust diagnostic power. Expression of the genes was also correlated with key FF metabolic and endocrine parameters in the PCOS group, including an inverse association between *PNPLA2* and triglycerides, with concomitant positive correlations of *G0S2* and *HILPDA* with insulin and testosterone, respectively. Moreover, in vitro androgen exposure recapitulated major features of lipolytic dysregulation in normal CCs, highlighting a direct mechanistic role of androgen excess in suppressing lipolysis.

Lipid metabolism impairment in GCs is an important characteristic of PCOS, which significantly contributes to the disease's progression [19]. A growing body of evidence obtained from high‐throughput transcriptome sequencing of PCOS GCs has identified numerous differentially expressed mRNAs enriched in metabolic processes relating to lipids, particularly fatty acid biosynthesis, lipid transportation, and the peroxisome proliferator‐activated receptor (PPAR) signaling pathway [6, 7, 8], indicating transcriptional dysregulation of lipid metabolism in GCs from women with PCOS. Consistent with these findings, we observed significant alterations in mRNA levels of genes playing a leading role in the mobilization of lipid droplets. Of note, while *PNPLA2* was downregulated in PCOS CCs, transcript levels of *G0S2* and *HILPDA* were increased, accompanied by a borderline decline in *ABHD5* expression.

ATGL is a key enzyme that mediates the initial step of triglyceride hydrolysis, producing diacylglycerol and a free fatty acid. The *PNPLA2* gene is responsible for encoding ATGL. One of the transcription factors that makes a substantial contribution to *Pnpla2* expression is forkhead box protein O1 (FOXO1), which induces *Pnpla2* transcription by directly interacting with its response element on the gene promoter [15]. PCOS was associated with diminished expression and transactivating performance of FOXO1 in ovarian follicular cells from both mice and humans, with insulin and dihydrotestosterone reproducing the same pattern in immortal human GCs [20, 21], suggesting that *PNPLA2* may be subjected to downregulation in PCOS GCs. In parallel with this deduction, our results demonstrated attenuated transcript abundance of *PNPLA2* in CCs from PCOS patients, with attendant increased testosterone levels in FF. In addition, testosterone dose‐dependently downregulated *PNPLA2* in cultured CCs, reaffirming that *PNPLA2* expression may be suppressed in PCOS CCs, likely owing to hyperandrogenism‐triggered inhibition of FOXO1 function. By contrast, a previous study has found elevated *Pnpla2* expression in the ovaries of rats prenatally exposed to androgen excess [22]. Nevertheless, the contribution of *Pnpla2* transcripts from cells other than CCs, especially oocytes, the stage‐specific effect of androgens on follicles, and compensatory epigenetic alterations in response to prenatal exposure might underlie such a contradiction.

The full enzymatic activity of lipid droplet‐associated ATGL necessitates its interaction with the protein coactivator ABHD5 [15]. Although transcriptional regulation of *ABHD5* is yet to be completely elucidated, the transcription factors cAMP‐response element binding protein (CREB) and zinc finger E‐box binding homeobox 1 (ZEB1) have been demonstrated to serve as important inducers of *ABHD5* expression [23]. There is evidence indicating a decrement in CREB function and *ZEB1* transcription in GCs from women with PCOS [24, 25], which may favor a possible reduction in *ABHD5* transcription. Likewise, we observed that PCOS was associated with a modest *ABHD5* downregulation in CCs. However, our study showed no noticeable change in *ABHD5* expression in CCs treated with testosterone concentrations found in the intrafollicular milieu of PCOS patients, suggesting that the relative decrease of *ABHD5* expression in PCOS CCs may result from dysregulation of factors other than androgens, such as microRNAs, cytokines, and metabolites. In support of this, recent research has revealed that mRNA abundance of *ABHD5* in HepG2 cells was not affected by high doses of testosterone alone but was significantly altered following treatment with testosterone and free fatty acids in combination [26].

Despite participation in a variety of processes, including cell cycle regulation, mitochondrial respiration, and apoptosis, G0S2 is mainly characterized by its function as a non‐competitive inhibitor of ATGL [15]. The *G0S2* gene contains a functional binding sequence for PPARs in its promoter region, and PPARγ is among the major transcriptional regulators of *G0S2* [27]. It has been reported that PPARγ expression was increased in GCs from women with PCOS [28] and dose‐dependently promoted by dihydrotestosterone in rat GCs in vitro [29], implying a probable link between PCOS and *G0S2* upregulation mediated by hyperandrogenism. This partly concurs with our findings, as we noted markedly elevated *G0S2* expression in CCs from PCOS patients, though transcript levels of *G0S2* were neither correlated with testosterone concentrations in FF nor influenced in cultured CCs exposed to high doses of testosterone. Instead, our results revealed a positive correlation between *G0S2* expression and intrafollicular amounts of insulin in women with PCOS, indicating that *G0S2* upregulation in PCOS CCs may be underlain by hyperinsulinemia. Concurrently, *G0S2* has been demonstrated to be induced under hyperinsulinemic conditions, with no concomitant alteration in *PNPLA2* and *ABHD5* [30]. Interestingly, expression of neither *PNPLA2* nor *ABHD5* in CCs was correlated with FF insulin concentrations in our study.

Aside from G0S2, ATGL activity is inhibited by another lipid droplet‐associated peptide coregulator called HILPDA. As its name suggests, HILPDA is highly induced during hypoxia due to the binding of hypoxia‐inducible factors (HIFs) to multiple hypoxia response elements located in the promoter of the *HILPDA* gene [15]. HIF‐1α has been shown to be highly expressed in ovarian tissue and GCs from PCOS rats and women, respectively, with dihydrotestosterone treatment bringing about its upregulation in cultured GCs [31, 32], a pattern that can probably be the case for *HILPDA* transcription. In keeping with this postulation, we found increased *HILPDA* expression in CCs from women with PCOS. Notably, *HILPDA* was positively correlated with intrafollicular testosterone in the PCOS group, and exposure to testosterone concentrations comparable to those observed in PCOS follicles elevated its expression in CCs in vitro, supporting the role of androgen excess in PCOS‐driven upregulation of *HILPDA*.

Although the existing findings are somewhat contradictory, presumably at least in part owing to depot‐specific differences in adipose tissue function and regulation, the prevailing body of evidence obtained from both animal models and humans indicates that PCOS is associated with downregulation of genes encoding the lipolytic machinery, particularly canonical lipases, in adipose tissue [33, 34, 35]. Concordant with this molecular signature, the majority of relevant studies have shown that whole‐body lipolysis is reduced in PCOS patients [36, 37, 38]. Despite these systemic observations, insights into localized intrafollicular lipolysis have remained lacking until now. Our results demonstrated a decline in *PNPLA2* expression in conjunction with the upregulation of *G0S2* and *HILPDA* in CCs from women with PCOS, an expression profile that accords with transcriptional suppression of lipolysis in CCs, given the cellular functions of the respective gene products. In agreement, we noted increased intracellular neutral lipids in PCOS CCs, phenotypically confirming the relationship between PCOS and inhibited lipolysis in these cells. Importantly, our in vivo findings were recapitulated to a great extent in cell culture models, such that CCs treated with high doses of testosterone displayed a decrease in mRNA levels of *PNPLA2*, along with elevations in *HILPDA* expression and lipid droplet content, implying that hyperandrogenism may substantially contribute to PCOS‐induced attenuation of lipolytic activity in CCs by dysregulating expression of key lipolysis‐related genes. We also found an inverse correlation between transcript abundance of *PNPLA2* in CCs and triglyceride concentrations in FF. One possible explanation for this association is negative feedback by increased intrafollicular triglyceride levels on the lipolytic function of CCs. Alternatively, given the ability of GCs to synthesize and release triglyceride‐rich lipoproteins [39], this relationship may reflect that triglycerides exceeding cellular storage capacity are secreted into FF as a protective mechanism against lipotoxicity.

Because lipid homeostasis is determined by the balance between lipid mobilization and lipid biosynthesis, augmented *de novo* lipogenesis may represent an additional mechanism contributing to intracellular neutral lipid accumulation observed in PCOS CCs, alongside defective lipolysis. Corroboratively, expression of numerous lipogenic genes, including PPARγ, sterol regulatory element‐binding protein 1 (*SREBP1*), acetyl‐CoA carboxylase 1 (*ACC1*), and fatty acid synthase (*FASN*), has been reported to be increased in GCs from women with PCOS [28]. More directly, it has been demonstrated that elevation of lipid droplet content in androgen‐treated GCs was associated with coincident downregulation of ATGL and upregulation of G0S2, SREBP1, PPARγ, ACC1, and FASN [40, 41], supporting the speculation that intracellular neutral lipid accumulation in PCOS GCs may arise from concerted suppression of lipolysis and activation of *de novo* lipogenesis.

While it has been documented that mammalian CCs contain plenty of lipid droplets and express main lipases, little is known about the biological importance of lipolysis in these cells. Evidence from women undergoing assisted reproduction suggests that mobilization of intracellular neutral lipids in CCs may be involved in nuclear maturation of oocytes, possibly by supplying fatty acids for β‐oxidation during late meiotic stages [42]. Furthermore, fatty acids derived from hydrolysis of lipid droplet‐stored triglycerides in CCs have been revealed to be transported into the oocyte, wherein they were used to biosynthesize intracellular neutral lipids indispensable for embryonic development [43]. Collectively, these observations indicate that the lipolytic activity of CCs may play a role in preparing the oocyte for maturation and fertilization. As elaborated earlier, our results support the idea of lipolysis impairment and the consequent excessive lipid droplet accumulation in PCOS CCs, in which androgen excess makes a significant mediating contribution. On the other hand, women with full‐blown PCOS and pertinent morbidities like obesity or insulin resistance tend to exhibit poorer oocyte quality as a whole [44]. Taking all of these into account, it can be hypothesized that PCOS‐triggered suppression of lipolysis in CCs may expose the oocyte to an insufficient supply of fatty acids required for metabolic energy production and intracellular neutral lipid storage, thereby representing a potential mechanistic basis for compromised oocyte competence in affected women. This notion aligns with our finding of a significantly diminished fertilization rate in the PCOS group. Nevertheless, it is noteworthy that there was no correlation between the expression of the lipolysis‐related genes in CCs and IVF outcomes. Apart from the biological heterogeneity of PCOS and the multifactorial nature of reproductive performance, the lack of data on embryo competence at later stages of pre‐implantation development in the present study may partly explain this discrepancy, as the reliance of embryonic cells upon stores of lipid droplets inherited from the oocyte is likely to become more pronounced at the blastocyst stage and beyond.

Some limitations need to be acknowledged in this study. Firstly, the overall sample size, especially in the assessment of intracellular neutral lipids in CCs, was relatively small. Secondly, expression at the protein level, post‐translational modifications, and protein–protein interactions were not evaluated, not allowing us to make conclusive deductions about functional and regulatory aspects of the genes of interest. Finally, since glycerol and free fatty acids, as direct products of lipolysis, have low molecular weights and may diffuse across the blood‐follicle barrier and freely enter the intrafollicular milieu, the lipolytic activity was assessed by measuring lipid droplet content as a surrogate biomarker, which may not specifically reflect the metabolic flux of lipolysis.

## 5 Conclusion

This study provides the first evidence that lipolysis is significantly inhibited in CCs from women with PCOS, manifested through *PNPLA2* downregulation, increased *G0S2* and *HILPDA* expression, and excessive intracellular neutral lipid accumulation. This altered transcriptional profile had a strong BMI‐independent association with PCOS and demonstrated notable discriminatory efficiency, while also correlating with key intrafollicular metabolic and endocrine parameters, most importantly testosterone. Additionally, the in vitro findings further support a contributory role of androgen excess in driving lipolytic dysregulation. Our data suggest that impaired lipolysis in CCs may limit fatty acid availability to the oocyte, potentially compromising its metabolic competence and fertilization capacity. Clinically, targeting androgen‐driven metabolic disturbances and restoring lipolytic balance in the follicular microenvironment may represent a promising strategy to improve oocyte quality and reproductive outcomes in women with PCOS. Future studies should prioritize larger cohorts, protein‐level validations, specific measurements of lipolytic flux, and direct examination of the interplay between lipolysis in CCs and oocyte competence.

## Funding

This work was supported by Tabriz University of Medical Sciences (Grant No. 66719).

## Disclosure

The authors have nothing to report.

## Ethics Statement

All subjects agreed to participate in the study by providing written informed consent. The whole research process was approved by the Ethics Committee of Tabriz University of Medical Sciences (Code: IR.TBZMED.REC.1400.037).

## Consent

The authors have nothing to report.

## Conflicts of Interest

The authors declare no conflicts of interest.

## Supporting information


**Table S1:** List of primers used for real‐time qPCR.
**Table S2:** Logistic regression analysis of the lipolysis‐related genes in association with the odds of PCOS.

## Data Availability

The data that support the findings of this study are available from the corresponding author upon reasonable request.
