# Supplementary material for: Androgen‐Driven Dysregulation of Lipolysis in Cumulus Cells: Molecular Evidence and Metabolic Implications in Polycystic Ovary Syndrome
Source: Reprod Med Biol. 2026 Aug 31;25(1):e70093. doi: 10.1002/rmb2.70093 (PMC13530396; doi:10.1002/rmb2.70093)
Supplement: Supplementary file 1 [file RMB2-25-e70093-s001.docx]

# Supplementary material

**Table S1** List of primers used for real-time qPCR

| Gene | Species | Accession No. | Direction | Sequence (5′- 3′) | Product size (bp) |
| --- | --- | --- | --- | --- | --- |
| PNPLA2 | Human | NM_020376.4 | Forward | GCTTCCTCGGCGTCTACTAC | 165 |
|  |  |  | Reverse | CAATGAACTTGGCACCAGCC |  |
| ABHD5 | Human | NM_016006.6 | Forward | AGACCCAGGTTTGACAGTGATG | 139 |
|  |  |  | Reverse | AGTAAGCAGCAGCCAAGAATCC |  |
| G0S2 | Human | NM_015714.4 | Forward | CGTGCCACTAAGGTCATTCC | 129 |
|  |  |  | Reverse | GCACGTACAGCTTCACCATC |  |
| HILPDA | Human | NM_013332.4 | Forward | AAGCATGTGTTGAACCTCTACC | 144 |
|  |  |  | Reverse | TGTGTTGGCTAGTTGGCTTCT |  |
| GAPDH | Human | NM_002046.7 | Forward | GCATCCTGGGCTACACTGA | 162 |
|  |  |  | Reverse | CCACCACCCTGTTGCTGTA |  |

**Table S2** Logistic regression analysis of the lipolysis-related genes in association with the odds of PCOS

|  | B (SE) | OR [95% CI] | *p* |
| --- | --- | --- | --- |
| *Unadjusted single-gene models* | | | |
| PNPLA2 | -1.491 (0.619) | 0.225 [0.067, 0.757] | 0.016 |
| Intercept | 1.583 (0.700) | 4.868 | 0.024 |
| logit(*P*(PCOS)) = 1.583 – 1.491 PNPLA2 | | | |
| R^2^ = 0.149 |  |  |  |
|  |  |  |  |
| ABHD5 | -0.994 (0.552) | 0.370 [0.125, 1.092] | 0.072 |
| Intercept | 1.003 (0.612) | 2.726 | 0.102 |
| logit(*P*(PCOS)) = 1.003 – 0.994 ABHD5 | | | |
| R^2^ = 0.077 |  |  |  |
|  |  |  |  |
| G0S2 | 1.196 (0.481) | 3.308 [1.289, 8.486] | 0.013 |
| Intercept | -1.601 (0.698) | 0.202 | 0.022 |
| logit(*P*(PCOS)) = –1.601 + 1.196 G0S2 | | | |
| R^2^ = 0.150 |  |  |  |
|  |  |  |  |
| HILPDA | 0.730 (0.271) | 2.075 [1.219, 3.532] | 0.007 |
| Intercept | -1.351 (0.555) | 0.259 | 0.015 |
| logit(*P*(PCOS)) = –1.351 + 0.730 HILPDA | | | |
| R^2^ = 0.199 |  |  |  |
|  |  |  |  |
| *Single-gene models adjusted for age and BMI* | | | |
| PNPLA2 | -1.559 (0.637) | 0.210 [0.060, 0.733] | 0.014 |
| Age | -0.030 (0.062) | 0.970 [0.860, 1.095] | 0.626 |
| BMI | -0.020 (0.095) | 0.980 [0.814, 1.180] | 0.833 |
| Intercept | 3.093 (2.782) | 22.044 | 0.266 |
| logit(*P*(PCOS)) = 3.093 – 1.559 PNPLA2 – 0.030 Age – 0.020 BMI | | | |
| R^2^ = 0.157 |  |  |  |
|  |  |  |  |
| ABHD5 | -1.029 (0.565) | 0.357 [0.118, 1.081] | 0.068 |
| Age | -0.011 (0.059) | 0.989 [0.882, 1.110] | 0.853 |
| BMI | 0.030 (0.091) | 1.030 [0.863, 1.230] | 0.743 |
| Intercept | 0.602 (2.412) | 1.826 | 0.803 |
| logit(*P*(PCOS)) = 0.602 – 1.029 ABHD5 – 0.011 Age + 0.030 BMI | | | |
| R^2^ = 0.079 |  |  |  |
|  |  |  |  |
| G0S2 | 1.232 (0.486) | 3.427 [1.322, 8.886] | 0.011 |
| Age | -0.025 (0.060) | 0.975 [0.866, 1.097] | 0.673 |
| BMI | -0.019 (0.093) | 0.981 [0.818, 1.177] | 0.839 |
| Intercept | -0.383 (2.520) | 0.682 | 0.879 |
| logit(*P*(PCOS)) = –0.383 + 1.232 G0S2 – 0.025 Age – 0.383 BMI | | | |
| R^2^ = 0.156 |  |  |  |
|  |  |  |  |
| HILPDA | 0.813 (0.294) | 2.255 [1.268, 4.010] | 0.006 |
| Age | 0.052 (0.063) | 1.053 [0.930, 1.193] | 0.412 |
| BMI | 0.012 (0.093) | 1.012 [0.844, 1.213] | 0.899 |
| Intercept | -3.392 (2.840) | 0.034 | 0.232 |
| logit(*P*(PCOS)) = –3.392 + 0.813 HILPDA + 0.052 Age + 0.012 BMI | | | |
| R^2^ = 0.214 |  |  |  |
|  |  |  |  |
| *Unadjusted multi-gene model* | | | |
| PNPLA2 | -1.958 (0.702) | 0.141 [0.036, 0.559] | 0.005 |
| G0S2 | 1.515 (0.576) | 4.548 [1.470, 14.070] | 0.009 |
| HILPDA | 0.826 (0.349) | 2.285 [1.153, 4.528] | 0.018 |
| Intercept | -1.442 (1.086) | 0.236 | 0.184 |
| logit(*P*(PCOS)) = –1.442 – 1.958 PNPLA2 + 1.515 G0S2 + 0.826 HILPDA | | | |
| R^2^ = 0.455 |  |  |  |
|  |  |  |  |
| *Multi-gene model adjusted for age and BMI* | | | |
| PNPLA2 | -2.022 (0.731) | 0.132 [0.032, 0.555] | 0.006 |
| G0S2 | 1.598 (0.611) | 4.944 [1.492, 16.388] | 0.009 |
| HILPDA | 0.795 (0.364) | 2.214 [1.084, 4.520] | 0.029 |
| Age | -0.012 (0.073) | 0.988 [0.856, 1.141] | 0.870 |
| BMI | -0.042 (0.110) | 0.959 [0.773, 1.190] | 0.705 |
| Intercept | 0.020 (3.388) | 1.020 | 0.995 |
| logit(*P*(PCOS)) = 0.020 – 2.022 PNPLA2 + 1.598 G0S2 + 0.795 HILPDA – 0.012 Age – 0.042 BMI | | | |
| R^2^ = 0.459 |  |  |  |

R² values represent Nagelkerke’s pseudo R².

B, unstandardized log-odds coefficient; BMI, body mass index; CI, confidence interval; OR, odds ratio; SE, standard error
